# Using a standalone ear-EEG device for focal-onset seizure detection

**DOI:** 10.1186/s42234-023-00135-0

**Published:** 2024-02-07

**Authors:** McGregor Joyner, Sheng-Hsiou Hsu, Stephanie Martin, Jennifer Dwyer, Denise Fay Chen, Reza Sameni, Samuel H. Waters, Konstantin Borodin, Gari D. Clifford, Allan I. Levey, John Hixson, Daniel Winkel, Jonathan Berent

**Affiliations:** 1 NextSense Inc., Mountain View, CA USA; 2 Department of Neurology, Emory University School of Medicine, Atlanta, GA USA; 3 Department of Biomedical Engineering, Georgia Institute of Technology, Atlanta, GA USA; 4 Department of Neurology, University of California San Francisco, San Francisco, CA USA; 5 Department of Biomedical Informatics, Emory University School of Medicine, Atlanta, GA USA

**Keywords:** Ear-EEG, Long-term EEG, Focal epilepsy, Seizure detection, Temporal lobe, Wearable technologies

## Abstract

**Background:**

Seizure detection is challenging outside the clinical environment due to the lack of comfortable, reliable, and practical long-term neurophysiological monitoring devices. We developed a novel, discreet, unobstructive in-ear sensing system that enables long-term electroencephalography (EEG) recording. This is the first study we are aware of that systematically compares the seizure detection utility of in-ear EEG with that of simultaneously recorded intracranial EEG. In addition, we present a similar comparison between simultaneously recorded in-ear EEG and scalp EEG.

**Methods:**

In this foundational research, we conducted a clinical feasibility study and validated the ability of the ear-EEG system to capture focal-onset seizures against 1255 hrs of simultaneous ear-EEG data along with scalp or intracranial EEG in 20 patients with refractory focal epilepsy (11 with scalp EEG, 8 with intracranial EEG, and 1 with both).

**Results:**

In a blinded, independent review of the ear-EEG signals, two epileptologists were able to detect 86.4% of the seizures that were subsequently identified using the clinical gold standard EEG modalities, with a false detection rate of 0.1 per day, well below what has been reported for ambulatory monitoring. The few seizures not detected on the ear-EEG signals emanated from deep within the mesial temporal lobe or extra-temporally and remained very focal, without significant propagation. Following multiple sessions of recording for a median continuous wear time of 13 hrs, patients reported a high degree of tolerance for the device, with only minor adverse events reported by the scalp EEG cohort.

**Conclusions:**

These preliminary results demonstrate the potential of using ear-EEG to enable routine collection of complementary, prolonged, and remote neurophysiological evidence, which may permit real-time detection of paroxysmal events such as seizures and epileptiform discharges. This study suggests that the ear-EEG device may assist clinicians in making an epilepsy diagnosis, assessing treatment efficacy, and optimizing medication titration.

**Supplementary Information:**

The online version contains supplementary material available at 10.1186/s42234-023-00135-0.

## Background

Epilepsy is a chronic neurological disorder that is characterized by recurrent and mostly unpredictable seizures. Timely diagnosis and proper treatment, however, can lead to seizure-free lives for up to 70% of individuals living with epilepsy. Unfortunately, out of an estimated 50 million individuals living with epilepsy worldwide, around 80% reside in low- and middle-income countries with little access to proper treatment (World Health Organization 2019). Electroencephalography (EEG) has long been the gold standard for diagnosing and characterizing epilepsy, when coupled with a nuanced clinical history and correlative video evidence. However, the paroxysmal nature of these events presents various barriers to their clinical detection. Routine EEG is limited by the sparsity of events captured during brief recordings and can be non-diagnostic in about 50% of cases (Smith 2005; McGinty et al. 2019). Ambulatory EEG, in contrast, offers the benefit of prolonged recording (> 48–72 hrs), but in practice introduces technical challenges that make it similarly prone to yielding non-diagnostic results (Worrell et al. 2002; Faulkner et al. 2012; Seneviratne et al. 2013)*.* Likewise, alternative biosignal modalities used in ambulatory seizure characterization yield low sensitivity or are prone to a high rate of false positive detection (Ryvlin et al. 2020). Therefore, neurologists and epileptologists often are forced to rely on self-reports by patients to craft their diagnosis and treatment plans, lacking reliable neurophysiological confirmation from population-wise studies. Unfortunately, seizure diaries have been shown to be highly inaccurate, thus undermining the scientific basis for treatment of the disease (Fisher et al. 2012). Patients themselves have been known to fail to report almost three quarters of the complex partial seizures they experience and almost 90 % of all seizures that occur while sleeping (Hoppe et al. 2007). Thus, despite a variety of challenges encumbering clinical EEG, it has remained the gold standard alongside video evidence in the absence of more reliable and accessible methods.

In most cases, a non-invasive, scalp-mounted electrode montage (scalp EEG) is sufficient to perform routine or ambulatory EEG. However, noninvasive EEG is not always conclusive to the diagnosis and characterization of epilepsy. Intracranial EEG is an alternative procedure for brain monitoring that involves implanting electrodes inside the skull. By recording electrical signals closer to the sources of pathology, and with superior three-dimensional sampling, intracranial EEG allows for more precise monitoring and localization of epileptiform and ictal activity. The electrodes can be implanted in various brain regions to locate the origin of seizures and map the neural activity associated with them. This information guides treatment options such as surgical planning or the consideration of neurostimulation devices. However, it is an invasive procedure that requires surgical electrode placement, which can in turn lead to complications such as infection or bleeding, and is therefore reserved for clinical cases where non-invasive methods have failed or when a high degree of precision is required for diagnosis or treatment approaches.

Given the practical challenges encumbering scalp and intracranial EEG, the outer ear offers an alternative location from which to access cerebral activity with high fidelity and stability (Ne et al. 2021). Specifically, the external ear canal and cymba conchae are anatomically close to the temporal lobe, which is commonly implicated in the onset or propagation of focal seizures. Additional evidence suggests that electrodes placed at the opening of the ear canal might be less prone to signal attenuation for neural sources in the temporal region than those distributed about the scalp (Yarici et al. 2023). Recent reviews have enumerated the myriad of physiological signals and health phenomena that can be captured using sensors in and around the outer ear (Ne et al. 2021; Röddiger et al. 2022; Bleichner and Debener 2017). For instance, prior research has already demonstrated the feasibility of using the ear as a recording site not only for electrographic seizures (Zibrandtsen et al. 2017; Zibrandtsen et al. 2018), but also for other spontaneous, evoked, or induced neurological activity, such as brain waves associated with sleep (Mikkelsen et al. 2019), posterior dominant alpha rhythms during eyelid closure (Mikkelsen et al. 2015; Kappel et al. 2019; Kaveh et al. 2020), steady-state visual evoked potentials (Kwak and Lee 2020) and auditory brain responses (Christensen et al. 2018). Several studies have even demonstrated the effectiveness of automated algorithmic methods when applied to in-ear or behind-the-ear EEG for epilepsy use cases (Gu et al. 2017; Vandecasteele et al. 2020; You et al. 2022).

**Fig. 1:**
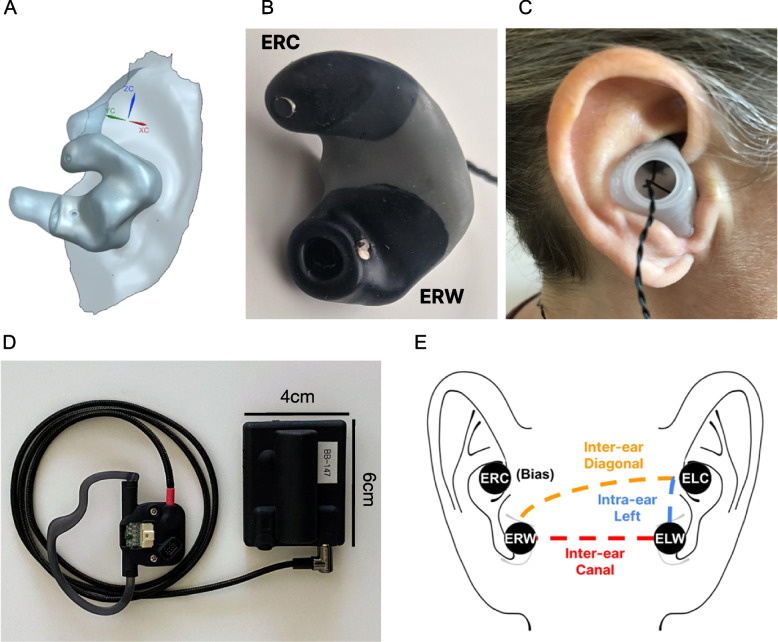
Ear-EEG system. **a** 3D anatomical ear model obtained through optical ear scan. **b** A custom-made earbud with two electrodes per ear – Ear Right Cymba (ERC) placed in the cymba conchae and Ear Right Whole Canal (ERW) placed in the canal. **c** Non-invasive in-ear fit of the earbud. **d** Data acquisition system consists of the “earboard” (left) hooked around the left ear and a data recording device (right). **e** The electrodes’ placement, bipolar channel configuration, and naming convention

In light of the electrographic recording potential of the ear and the limitations of both the scalp and intracranial EEG modalities, we developed a novel, discreet, comfortable, and non-invasive wearable device resembling a pair of wired earbuds (see Fig. 1B-D). The current study was conducted to directly compare the seizure detection efficacy of the ear-EEG recording modality with that of EEG recording modalities currently used in the clinical diagnostic environment. Although scalp EEG with video evidence (video EEG) is sufficient for the majority of patients, we will refer to both scalp EEG and intracranial EEG collectively as the “gold standard” EEG modalities against which the ear-EEG device is evaluated in this context. This study also aims to characterize how the seizure detection sensitivity of ear-EEG varies with focal seizure types and anatomic boundaries. We hypothesized that the electrodes used in the ear-EEG recording modality would (1) prove most sensitive to temporal lobe seizures and would (2) yield complementary, if not superior, signal quality to the traditional scalp montage, due to the aforementioned anatomical advantages of the recording sites (Yarici et al. 2023). We also present a supplementary analysis characterizing the effect of the EEG recording modality on the precise timing of seizure annotation onset and offset.

## Methods

### Ear-EEG system

The ear-EEG system used in this study has been designed to provide highly reliable neurological signals while optimizing user comfort and patient compliance (Fig. 1). Two earbuds are worn in-ear (Fig. 1B-C) and attached to a data-acquisition system (Fig. 1D). To promote comfort and wearability, the earbuds were manufactured from soft, biocompatible silicone material and shaped according to high-resolution anatomical scans of each patient’s ears (Fig. 1A; see “Custom earbuds” in the Results section). An expert in ear morphology with experience in custom earbud design subsequently provided optimal locations for EEG sensors by analyzing each digital model. The deleterious effects of acoustic occlusion and the advantages of open-fit or partially occlusive earpieces are well documented in the hearing aid literature (Winkler et al. 2016). Fortunately, incremental and diminishing amelioration of the occlusion effect has been described for hollow earpieces with a vent diameter of up to 3 mm (Kuk et al. 2005). We therefor designed our ear-EEG earbuds to have a hollow tube with a minimum diameter of 3 mm.

Electrodes were made of silver rivets and coated with a proprietary conductive polymer-based coating (Hendricks et al. 2020). There are two electrodes per earbud (Fig. 1B), placed in the cymba conchae (ExC) and in the canal (ExW). Given the form factor of the device, these two electrode positions were selected to provide optimal stability and contact surface area for the electrode-skin interface. The earbuds are connected to a proprietary analog-digital data acquisition system (Fig. 1D) consisting of (1) an “earboard” that hooks around the left ear, capable of amplifying, filtering, and digitizing up to eight EEG channels and triaxial accelerometry with a sampling rate of 500 Hz, and (2) a data recording device with an integrated micro-SD memory card and a battery lasting around 40 hrs from a single charge. In this study, four electrodes were placed as shown in Fig. 1E. In order to avoid having a single point of failure using a single reference electrode, the ear-EEG device digitizes and amplifies the voltage difference between each pair of electrodes (excluding the bias electrode ERC) to form a total of three bipolar channels. This configuration results in one intra-ear channel (ELW - ELC) and two channels that span electrodes on both ears: inter-ear canal (ERW - ELW) and inter-ear diagonal (ERW - ELC). We expected the available inter-ear channels to be useful for observing laterally differential activity with high sensitivity, while the intra-ear channel was expected to elucidate activity more local to the left temporal lobe (Yarici et al. 2023). The inter-ear diagonal channel provides largely redundant information and has been omitted from the figures presented here. The Emory University School of Medicine Institutional Review Board approved the use of the ear-EEG system for this study with a Nonsignificant Risk label.

### Participants and data acquisition

#### Cohorts

Between August 2019 and June 2021, 29 patients were admitted to the Emory Epilepsy Monitoring Unit (EMU) within the Emory University Hospital for presurgical or diagnostic evaluation and were subsequently enrolled in an ongoing observational study in the clinical setting. For the purpose of this research, simultaneous electrographic recordings were captured by an ear-EEG system alongside the gold standard EEG recordings already required for clinical evaluation. As part of the same study protocol (see section titled “Ethics approval and consent to participate”), several patients admitted to the Emory Sleep Center for polysomnography were enrolled in a similar research initiative regarding sleep characterization, which will not be discussed as part of this interim analysis. According to the protocol, patients were excluded in the event that they were under 18 years of age, unable to safely tolerate placement of earbuds (e.g., antecedent skin breakdown, recent injury to ear) or, in the case of participants undergoing scalp EEG monitoring, could not have all 16 non-midline surface electrodes placed. Patient enrollment was further impacted by voluntary withdrawal (*n* = 5), cancellation of planned clinical monitoring (*n* = 3), and one (*n* = 1) case of excessive earwax which prevented fitment of the ear-EEG system (see “Custom earbuds”, below). This loss in enrollment (*n* = 9) resulted in a dataset representing a total of 20 patients (9 males, 11 females, between 20 and 49 years of age, average ± STD 31.2 ± 8.95). See Supplementary Table S1, Additional File 1 for patient-wise recording and seizure information.

During the first phase of the experiment, nine patients underwent intracranial EEG recording. A subsequent cohort of 12 patients underwent scalp EEG recording during the second phase of the experiment. One patient originally recruited in the scalp EEG cohort returned for intracranial recording and was therefore included in both cohorts. Each admission to the EMU typically involved several days of clinical monitoring, allowing for multiple recording sessions per patient. We collected a total of 1255 hrs of ear-EEG over 106 sessions, with a median wear time of 13.0 hrs (see Tables 1 and S2 for a data summary).

**Table 1:** Summary of data acquisition and recording statistics

Reference modality	Intracranial EEG	Scalp EEG	Combined
Total # patients	9	12	20^†^
Total # sessions	62	44	106
Total # ground-truth seizures	30	26	56
Total recording time (hr)	571.4	683.6	1255
Mean recording time per session (hr)	9.2	15.5	11.8
Min recording time of a session (hr)	1	1.5	1
Max recording time of a session (hr)	23.5	23.8	23.8

^†^Note that one patient underwent both intracranial and scalp EEG recording sessions

#### Intracranial EEG

Intracranial EEG signals were obtained using stereo-EEG depth electrodes (DIXI Medical, Besancon, France), consisting of 5 to 18 platinum–iridium electrodes, with an inter-electrode distance of 3.5 mm. Electrode placement and implantation duration were dictated by clinical evaluation requirements. These signals were recorded bedside using the Quantum LTM Amplifier (Natus Inc., CA, USA), with a 2048 Hz sampling rate and up to 276 channels per patient. Subgaleal electrode contacts distant from epileptic foci and areas of interest were used for reference and grounding.

#### Scalp EEG

Scalp EEG signals were captured using the Xltek Brain Monitor EEG Amplifier (Natus Inc., CA, USA) at a 256 Hz sampling rate, following the international standard 10–20 system with 22 channels and FCz as the reference electrode.

#### Custom earbuds

During routine intracranial or scalp EEG recording sessions, technicians fitted custom-made earbuds to the patients’ ears for simultaneous recording, enabling direct comparison of signals across recording modalities. The 3D geometry of each custom-made earbud was determined using the ear-specific optical scanner eFit (United Sciences, GA, USA). Prior to initiating data collection and after custom fitment of the earbuds, technicians assessed the effect of the device on each patient’s hearing. Although a systematic psychoacoustic assessment was not deemed necessary in the context of this study, technicians asked patients to confirm that they felt capable of interacting normally with their environment (e.g., carrying a conversation at a normal volume, listening to music or television at a normal volume). A small amount of conductive paste was applied to the earbuds’ electrodes for contact quality and stability during long-term recording. At discharge, each patient completed an exit questionnaire evaluating the comfort of the ear-EEG device on a scale of 1 to 10 (1 being “Painful”, 10 being “Very Comfortable”).

#### Data alignment and preprocessing

The ear-EEG signals were high-pass filtered at 0.5 Hz with a fourth-order Butterworth filter (applied forward and backward) in order to remove DC drift before seizure annotation. To enable retrospective time alignment of the ear-EEG recordings with the gold standard EEG, we constructed an in-house “synchronization box.” This device generates an analog pseudo-random pulse-train “sync” signal, with a pulse width of 5 ms and a minimum pulse interval of 100 ms (i.e., a pulse frequency of approximately 10 Hz). The sync signal was transmitted simultaneously to both the Natus EEG amplifier and the ear-EEG system using touch-proof electrode connectors. This technique enabled the time alignment of the gold standard EEG and the ear-EEG with an error of less than 2–3 ms (i.e., 1–2 samples). Technical details of the algorithms for data alignment are provided in an additional file [see “Algorithm for time alignment of ear-EEG and gold standard EEG”, Additional File 1].

### Seizure annotations

#### Annotation procedure

Annotation of electrographic seizures from each recording modality (i.e., scalp, intracranial, or ear-EEG) was performed by two board-certified epileptologists. Each epileptologist (i.e., reviewer) had limited prior experience in reviewing ear-EEG signals for seizure activity but was asked to use their best judgment based on their training and experience in reviewing gold standard EEG. Specifically, reviewers were asked to annotate the onset and offset of every electrographic ictal period (i.e., seizure) based solely on electrographic signals using Natus NeuroWorks EEG Software. The reviewers annotated based purely on the visual identification of an electrographic seizure, using their clinical judgment, which is standard for any form of EEG review. Reviewers were also asked to report segments where excessive artifacts were evident, including flat line and saturated signals indicating loss of electrode contact.

**Fig. 2:**
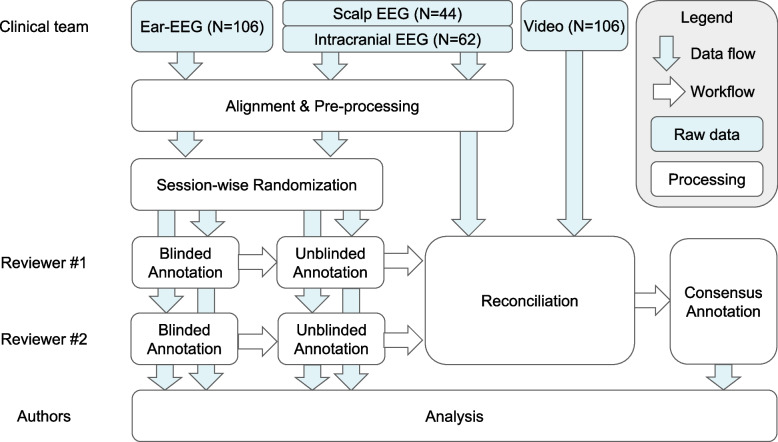
Annotation workflow. An illustration of the seizure annotation and analysis workflow from ear-EEG and gold standard EEG (intracranial or scalp)

In the blinded annotation process shown in Fig. 2, each reviewer was first asked to annotate the 62 sessions of ear-EEG from patients in the intracranial cohort in a randomized order without reference to the intracranial EEG signals. Next, the same two reviewers performed the unblinded annotation of intracranial EEG recordings from the same 62 sessions in a separately randomized order. Following completion of the sessions from the intracranial cohort, the reviewers continued to perform the blinded annotation and unblinded annotation in a similar fashion for the 44 sessions for which scalp EEG was the gold standard EEG modality. All annotations were performed for intracranial sessions before scalp sessions because the scalp EEG recordings were collected in the latter half of the clinical study. The only exception was Patient 20, who returned for intracranial implantation and recording after their scalp data had been collected and annotated.

#### Annotation reconciliation and exclusion criteria

In the reconciliation process shown in Fig. 2, a consensus seizure annotation was derived for each session from the two unblinded annotations made using the gold standard signal data. Agreement between both reviewers was required to include a seizure in the consensus annotations. In the first pass of the unblinded annotations process, there was one seizure annotated by only one reviewer, and four sessions (comprising a total of seven seizures) were not reviewed by one of the reviewers, due to technical issues in recording the synchronization signal, which made the alignment of the multi-hour ear-EEG and standard EEG infeasible for the reviewer. These discrepancies were resolved between the two reviewers to reach the final consensus on the seizure annotations.

From this dataset of consensus annotations (*n* = 56), we excluded from further analysis any seizures that occurred when ear-EEG recordings were corrupted due to poor contact quality (*n* = 3), uninterpretable due to data alignment issues (*n* = 3), or unavailable due to the complete removal of earbuds after the onset of adverse events as described below (*n* = 6). See Supplementary Table S1 for a patient-wise count of seizures included in the analysis.

### Seizure classification

In order to enable more granular analysis of seizure annotation, we also categorized each seizure annotation based on its type, localization, recording condition, and duration (see Table 2). Seizure type (“subclinical”, “motor element”, “secondary generalization”) was determined using both clinical notes as well as reviewer annotations. Localization of each seizure was also noted manually during the unblinded annotation process. Participant wakefulness (i.e., awake or asleep) was determined using clinical notes, with the exception of two seizures made during intracranial recording for which the state of wakefulness was unknown. Duration was computed via consensus annotation and stratified by increments of 25 s (as in Vandecasteele et al. 2020).

**Table 2:** An overview of seizure types, localization, lateralization, and recording conditions

	Modality	Intracranial EEG	Scalp EEG
Overall	Total	24	20
Seizure type	Subclinical	5	1
	Motor element	10	0
	Secondary generalization	8	11
	Not clear	1	8
Localization	Temporal	22	6
	Frontal	2	1
	Parietal	0	2
	Not clear	0	11
Participant wakefulness	Awake	8	13
	Asleep	14	7
	Not clear	2	0
Duration	0–25 s	1	0
	25–50 s	2	2
	50–75 s	1	6
	75–100 s	6	5
	> 100 s	14	7

### Data analysis

The sample size of this study was determined by feasibility and the authors performed no formal sample size calculations. The analysis of this feasibility study was limited to descriptive statistics without significance calculations.

### Annotation analysis

We evaluated the performance of annotations made using ear-EEG by examining sensitivity, F1 score, positive predictive value (PPV), and false detection rate per 24 hrs, with reference to the gold standard annotations. Any two seizure annotations were deemed in agreement if they overlapped by at least 1 s (as in Halford et al. 2015). Accordingly, all seizure annotations were classified in detection terminology as either true positive (TP), false positive (FP), or false negative (FN), with the ear-EEG annotations as predictions and the gold standard annotations as the truth.

We also investigated the timing relationships of ear-EEG seizure annotations with their corresponding gold standard annotations. In the event that a reviewer annotated multiple seizure events on ear-EEG overlapping one seizure event on gold standard EEG, or multiple seizure events on gold standard EEG overlapping one seizure event on ear-EEG, the intermediate onsets and offsets were omitted from this analysis. The aforementioned scenarios occurred four times, and our analysis shows that it did not correlate with reviewer, modality, patient, or session. False negative results were not considered.

### Interrater agreement

In order to quantify the agreement of annotations between each of the reviewers, we analyzed interrater variability for each electrographic modality (scalp EEG, intracranial EEG, and ear-EEG) using several methods. First, we computed Cohen’s kappa for each modality, along with indices of bias and prevalence computed in accordance with (Byrt et al. 1993). This involved reducing every epoch of each recording to a binary label for each rater. Next, we addressed event-wise agreement by reducing every annotated seizure to an event that was either annotated by one reviewer, the other reviewer, or both. The event-wise reduction enabled us to compute the fraction of event agreement (FEA) and the fraction of event duration agreement (FEDA), as described in (Halford et al. 2015).

## Results

### Device tolerability

Patients wore the ear-EEG device repeatedly throughout the course of multiple recording sessions for an average of 11.8 hrs (min = 1.0, max = 23.8; Table 1) per session. The devices were well adopted by all patients, who rated their comfort at 7.5 ± 1.8 out of 10 (1 = “Painful”, 10 = “Very Comfortable). Of the 20 patients in this study, none reported serious adverse events (AE). Likewise, no patients reported a noticeable degradation in hearing. When prompted through a feedback survey, some patients (*n* = 4) reported one or more minor AEs, all of which were mild, expected, unrelated to device deficiencies, and resolved without intervention. Although some AEs resulted in the loss of ear-EEG data, as described above, all were of minor consequence to the patient and none necessitated initiation of corrective and preventive actions (CAPA). All AEs reported occurred during scalp EEG recording and involved minor forms of discomfort in the external ear, such as skin irritation (*n* = 2), otalgia (*n* = 1), and discomfort wearing the device (*n* = 2), which one patient reported as causing difficulty falling asleep (*n* = 1). Patients in the intracranial EEG cohort rated the comfort of the device at an average of 8 out of 10 and did not report AEs of any kind.

### Signal quality

Several examples of seizures captured during the course of this study are presented below. For visualization, all data was bandpass filtered between .5 and 70 Hz using a backward and forward fourth-order Butterworth filter, with sensitivities shown on the right side of each plotting window.

### Case study #1: true positive (versus intracranial EEG) during sleep

**Fig. 3:**
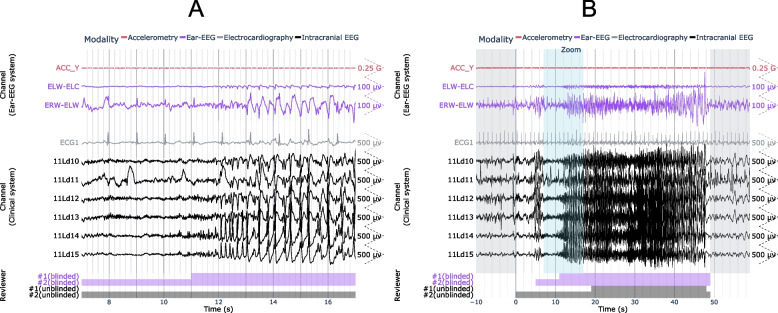
Example ear-EEG waveforms from a subclinical focal seizure detected during sleep. A 10 s window of interest is expanded in (**a**) and indicated in (**b**) by the “Zoom” region (blue), which falls within the full seizure annotation (white). Corresponding epileptologist annotations of seizure events are shown at the bottom of each plot, where the color-coded label “#1 (blinded)” refers to Reviewer #1’s annotation of the seizure made with access only to the ear-EEG system. ACC_Y: y-axis accelerometry from ear-EEG device; ELW-ELC: intra-ear left channel of ear-EEG; ERW-ELW: inter-ear canal channel of ear-EEG; ECG1: electrocardiogram channel; 11Ld10: the 10th recording site (equally spaced, with the 1st being deepest) along stereo-EEG depth electrode #11 placed on the left side of the head

Figure 3 shows a representative example of a subclinical temporal lobe focal seizure detected on ear-EEG during sleep. Both ear-EEG channels shown here measured stereotypical seizure waveforms and demonstrated clear seizure onset with evolution of electrographic patterns: low amplitude fast activity, sharp rhythmic theta, 2 Hz spike-and-wave activity, and finally postictal suppression. Similar ictal waveforms are observed in the intracranial EEG channels. The accelerometry channel (ACC_Y) confirmed the absence of confounding movements during this seizure.

### Case study #2: true positive (versus scalp EEG)

**Fig. 4:**
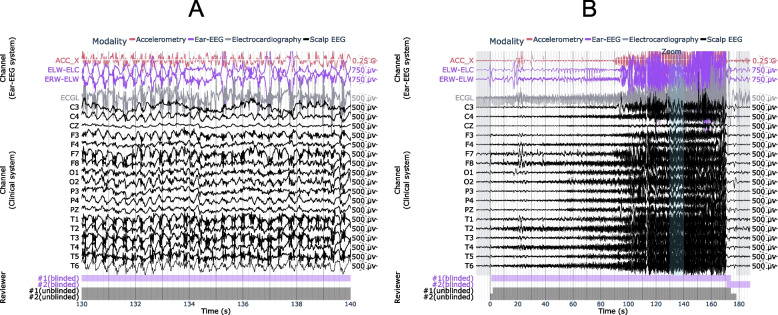
Example of focal-onset seizure detected on the ear-EEG and scalp EEG. A 10 s window of interest is expanded in (**a**) and indicated in (**b**) by the “Zoom” region (blue), which falls within the full seizure annotation (white). Corresponding epileptologist annotations of seizure events are shown at the bottom of each plot, where the color-coded label “#1 (blinded)” refers to Reviewer #1’s annotation of the seizure made with access only to the ear-EEG system. ACC_X: x-axis accelerometry from ear-EEG device; ECGL: electrocardiogram channel. Scalp EEG channels are labeled according to the International 10–20 system

Figure 4 shows a generalized, convulsive seizure that occurred while the patient was awake and was identified on both ear-EEG and scalp EEG. Characteristic spike and wave activity is clearly visible on both modalities, as seen 130 s into the seizure in Fig. 4A. The seizure can be observed on both the intra-ear left and inter-ear canal channels of the ear-EEG device.

### Case study #3: false negative and true positive (versus intracranial EEG)

**Fig. 5:**
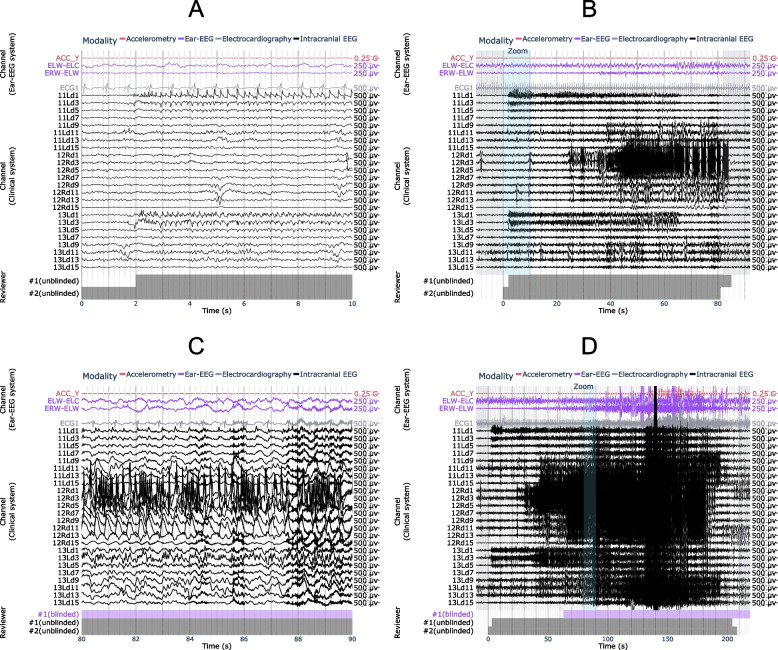
Examples of seizures from the same patient, with (**a**, **b**) false negative and (**c**, **d**) true positive results using ear-EEG. A 10 s window of interest is expanded in (**a**, **c**) and indicated in (**b**, **d**) by the “Zoom” region (blue), which falls within the full seizure annotation (white). Corresponding epileptologist annotations of seizure events are shown at the bottom of each plot, where the color-coded label “#1 (blinded)” refers to Reviewer #1’s annotation of the seizure made with access only to the ear-EEG system. ACC_Y: y-axis accelerometry from ear-EEG device; ECG1: electrocardiogram channel; 11Ld9: the 9th recording site (equally spaced, with the 1st being deepest) along stereo-EEG depth electrode #11 placed on the left side of the head

Figure 5A-B shows one of the seizures not detected on the ear-EEG, which emanated from deep structures within the mesial temporal lobe and did not propagate beyond its originating gyrus (parahippocampus). Figure 5C-D shows another example, with the same channels, of a seizure in the same patient that also emanated from within the mesial temporal lobe, but was captured by the ear-EEG as it propagated to the lateral temporal lobe.

### Case study #4: false positive (versus intracranial EEG) due to rhythmic movement

**Fig. 6:**
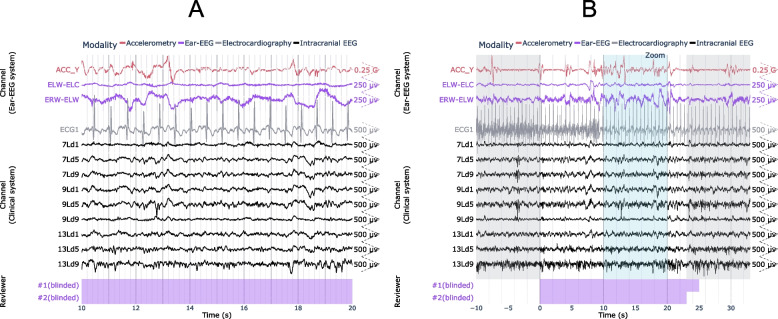
Example of false positive seizure identified on the ear-EEG. A 10 s window of interest is expanded in (**a**) and indicated in (**b**) by the “Zoom” region (blue), which falls within the full seizure annotation (white). Corresponding epileptologist annotations of seizure events are shown at the bottom of each plot, where the color-coded label “#1 (blinded)” refers to Reviewer #1’s annotation of the seizure made with access only to the ear-EEG system. ACC_Y: y-axis accelerometry from ear-EEG device; ECG1: electrocardiogram channel; 7Ld5: the 5th recording site (equally spaced, with the 1st being deepest) along stereo-EEG depth electrode #7 placed on the left side of the head

Figure 6 shows an example of a false positive annotation wherein a rhythmic signal appears to form in the inter-ear canal channel of the ear-EEG recording. Clinical notes from video evidence indicate that the patient was typing on their phone and proceeded to shake their head as if to gesture “no”. Close inspection of the accelerometry channel (ACC_Y) also indicates the presence of movement aligned with the artifact featuring prominently in the inter-ear canal channel.

### Interrater agreement

The interrater agreement analysis showed a high degree of concordance between reviewers on both gold standard modalities as well as on ear-EEG. Ear-EEG annotations were characterized by a Cohen’s kappa value of 0.77 (Table 3), along with agreement values of 68% for occurrence and 62% for duration (Table 4).

**Table 3:** Epoch-wise interrater agreement of seizure annotations by modality

Modality	Intracranial EEG	Scalp EEG	Ear-EEG
Cohen k	0.8419	0.9192	0.7662
Cohen k SE	0.0048	0.005	0.0054
Prevalence index	−0.9966	−0.9987	− 0.9982
Bias index	−0.0002	0	0

**Table 4:** Event-wise interrater agreement of seizure annotations by modality

Modality	Intracranial EEG	Scalp EEG	Ear-EEG
Events (#)	39	14	38
Events agreed (#)	22	14	26
FEA (%)	56.4	100	68.4
Events duration (s)	4041	1734	5022
Events agreed duration (s)	2939	1475	3120
FEDA (%)	72.7	85.1	62.1

*FEA* fraction of event agreement, *FEDA* fraction of event duration agreement

As shown in Table 3, the intracranial EEG annotations had more reviewer bias than either of the other modalities and lower Cohen’s kappa than scalp EEG annotations. Annotations made using ear-EEG signals yielded the lowest kappa value, with similar prevalence and bias indices to the scalp EEG annotations. The event-based agreement statistics shown in Table 4 also indicate the highest performance among scalp annotations on both fraction of event agreement (FEA) and fraction of event duration agreement (FEDA) metrics, although the sample size of scalp EEG annotations (see Table 1) was the smallest of the three modalities analyzed. Both reviewers agreed on all events annotated using scalp EEG, and the fraction of event agreement was higher for ear-EEG than it was for intracranial EEG. Like the kappa values reported in Table 3, the event duration analysis (FEDA) reflected the greatest agreement on scalp EEG, and the least agreement on ear-EEG (Table 4).

### Ear-EEG performance in detecting seizure events

Averaging results for both reviewers, 21 out of the 24 seizures identified on intracranial EEG had already been identified during blind annotation of the corresponding ear-EEG signals, representing an overall ear-EEG seizure detection sensitivity of 87.5% (see Table 5). Scalp EEG results were similar, with an average of 17 out of 20 ground-truth seizures (85%) detected by each reviewer on ear-EEG. All seizures not detected on ear-EEG (false negatives) were either subclinical (*N* = 2), had unclear origin (*N* = 2), or had motor elements (*N* = 2). The average F1 score across the entire dataset was 0.88. About 11% of the detections made on ear-EEG were false positives, which represented less than 0.1 per 24 hrs of recorded data, or about 1 false positive per 12 days of monitoring.

**Table 5:** Seizure detection performance of ear-EEG compared to gold standard EEG

Reference modality	Intracranial EEG			Scalp EEG			Combined		
Reviewer	1	2	Avg.	1	2	Avg.	1	2	Avg.
Reference seizures (count)	24	24	24	20	20	20	44	44	44
Sensitivity (%)	95.8	79.2	87.5	85	85	85	90.9	81.8	86.35
False Positive rate (per 24 hr)	0.084	0.21	0.147	0.035	0.035	0.035	0.057	0.115	0.086
F1 score	0.939	0.792	0.865	0.895	0.895	0.895	0.92	0.837	0.879
Positive Predictive Value (%)	92	79.2	85.6	94.4	94.4	94.4	93	85.7	89.35

We further evaluated the reviewers’ sensitivity for detecting seizures with ear-EEG given participant wakefulness as well as the seizure type, duration, and localization.

**Fig. 7:**
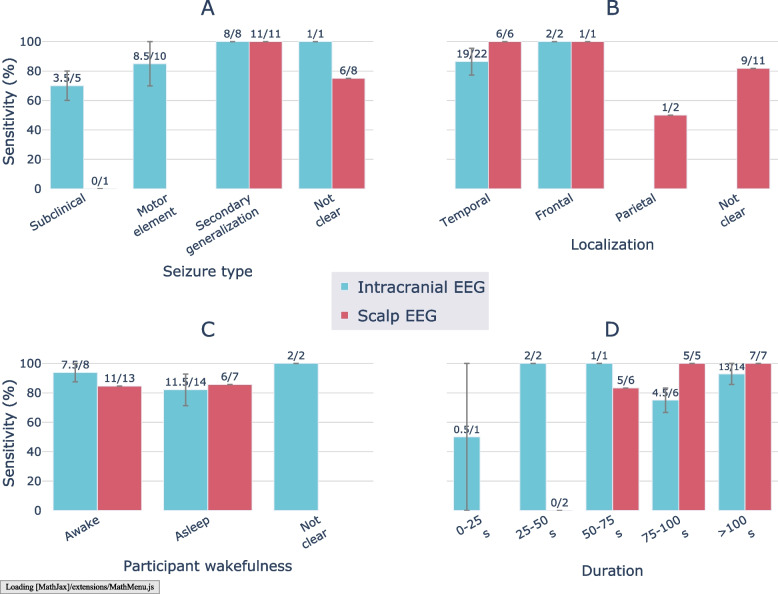
Sensitivity of seizure event detection on ear-EEG vs. gold standard EEG modalities by seizure types and recording conditions. Sensitivity values of the two reviewers are indicated by the colored bars (average) and the error bars (difference). Some categories were never observed in the dataset (e.g., Scalp EEG in Seizure type: Motor element)

As shown in Fig. 7A, all seizures with secondary generalization observed on intracranial (*N* = 8) and scalp (*N* = 11) EEG had been detected by the reviewers on ear-EEG. Conversely, the ear-EEG annotations failed to capture the only subclinical seizure observed on scalp EEG, while the reviewers averaged an ear-EEG sensitivity of 70% for the subclinical seizures observed on intracranial EEG (*N* = 5).

Figure 7B shows that seizures originating from the temporal or frontal lobe were detected on ear-EEG. A small number of frontal lobe seizures were observed on the scalp (*N* = 1) and intracranial (*N* = 2) recordings; all were detected on ear-EEG. Meanwhile, temporal lobe seizures had an ear-EEG sensitivity of 100% versus scalp EEG (*N* = 6) and 86.4% versus intracranial EEG (*N* = 22).

As shown in Fig. 7C, participant wakefulness did not appear to have a substantial effect on ear-EEG sensitivity. Duration of the seizure did not appear to have an effect on ear-EEG sensitivities for intracranial recordings, although the only seizures annotated on scalp EEG recordings that were not detected on ear-EEG (*N* = 3) had durations of less than 75 s.

### Timing difference in annotated seizure onset and offset detection

**Fig. 8:**
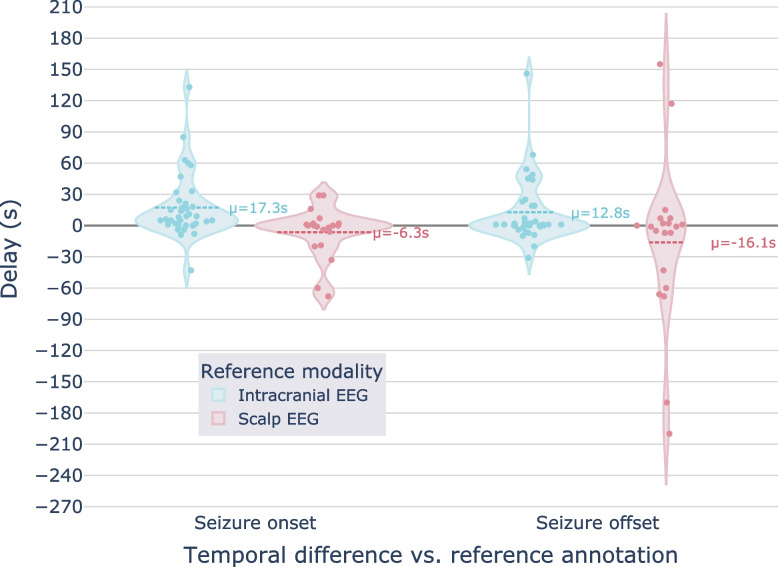
Time difference in seizure onset and offset detection of all seizures annotated on ear-EEG versus reference standard for intracranial EEG (blue) and scalp EEG (red). A positive delay means the seizure event annotated on ear-EEG lagged behind that on gold standard EEG

Figure 8 represents the onset and offset difference for each matching seizure event (seizure event timestamp on ear-EEG minus that of gold standard EEG). The timing relationship analysis indicated that seizure onsets were detectable earlier on intracranial EEG signals than on simultaneously recorded ear-EEG signals by an average of 17.3 s. However it is worth noting that five seizures were annotated on ear-EEG before the intracranial EEG. A similar effect was observed for seizure offsets, with an average delay of 12.8 s between intracranial EEG annotations and their matching ear-EEG annotations. The analysis results for the scalp EEG modality, however, indicated a slight *negative* bias in the delay, with ear-EEG annotations preceding those made using scalp EEG by an average of 6.3 s at seizure onset and 16.1 s at seizure offset.

## Discussion

In this study, we demonstrated a discreet, brain-sensing wearable device for long-term monitoring and detection of seizures, positioning it as a promising complement to conventional EEG monitoring systems. We comprehensively validated the seizure detection capacity of the ear-EEG system against clinical gold standard EEG (both intracranial and scalp EEG) in a clinical feasibility study consisting of 1255 hrs of simultaneous recordings from 20 patients.

In a blinded, independent review of the ear-EEG signals, two epileptologists achieved a reasonable event-wise interrater agreement of ear-EEG annotations (Cohen’s kappa = 0.77) and were able to detect 87.5% (intracranial) and 85.0% (scalp) of the seizures that were subsequently identified using the clinical gold standard modalities. The two reviewers noted that the ear-EEG signal contained stereotypical ictal waveforms such as low amplitude fast activity, sharp rhythmic theta, rhythmic spike-and-wave activity, and postictal suppression. All seizures not detected on the ear-EEG signals emanated from deep within the mesial temporal lobe or extra-temporally and remained very focal, without significant propagation. The reviewers attributed the low false positive rate observed in this study (0.1 per day) to the dynamical evolution of the seizure which was typically salient during review of the ear-EEG signal. It is worth noting that all patients with seizures had at least one event detected on ear-EEG, suggesting improved likelihood of detecting pathological events given the prolonged monitoring capability afforded by the device. These preliminary results demonstrate the feasibility of using an ear-EEG system to capture focal-onset seizures in long-term monitoring with accuracy that exceeds self-reported events (Fisher et al. 2012).

Further investigation of seizure types revealed that ear-EEG reliably captures temporal lobe seizures (86% for intracranial patients and 100% for scalp patients), which account for approximately 40% of all forms of epilepsy (Semah et al. 1998). This finding is consistent with observations previously made about the ear-EEG modality’s sensitivity to temporal lobe activity (Yarici et al. 2023), as well as our own hypothesis that ear-EEG would be more sensitive to temporal lobe seizures given the proximity of the earbuds to the inferior and lateral temporal lobes. It is worth noting that ear-EEG also captured all 3 frontal lobe seizures (100% sensitivity), another common seizure type trailing temporal lobe seizures. In addition, 100% of analyzed seizures with secondary generalization were detected on ear-EEG — this shows promise for capturing a large proportion of seizure types, including primary generalized seizures, which account for more than 40% of all epilepsies. The high sensitivity of seizure detection in these seizure types could also be attributed to the inter-ear referenced EEG channels used in the ear-EEG system (inter-ear canal and inter-ear diagonal, see Fig. 1E), which both maximize the distance between electrodes. Around 80% of all seizures occurring during sleep were successfully identified using the ear-EEG device, which may represent a solution to the majority of detections missing from the overnight portion of conventional seizure diaries.

The timing differences in seizure onset and offset detection suggest that the signal characteristics used by epileptologists to identify transitions in the evolution of a seizure become salient at different times depending on the recording modality reviewed. Given the increased spatial resolution and signal fidelity of the intracranial modality, seizures are expected to propagate to, and become observable on, intracranial electrodes prior to their extracranial appearance. Accordingly, the ear-EEG seizure annotations we analyzed had later onsets on average than intracranial annotations of the same event. An interesting observation from the analysis was that on average, seizure annotation onsets made using ear-EEG signals preceded annotation onsets of the same event made using scalp EEG. This observation further supports the hypothesis that the anatomical vantage point of the ear canals could be complementary, if not superior, to the analogous sensing locations of the scalp modality (e.g., T9, T10). We encourage further research to test this hypothesis and the degree to which this advantage generalizes.

Patients reported a high degree of tolerance for the device, underscoring its potential for widespread adoption. Only minor adverse events were reported for the scalp EEG cohort, who wore the device continuously for a median recording duration of 13.0 hrs. No intracranial patients reported adverse events. The enhanced patient comfort and non-intrusive nature of the ear-EEG device make it a compelling alternative to traditional EEG methodologies, which are fraught with practical challenges and limited accessibility. In addition to its advantageous form factor, the ear-EEG device provided reliable neurophysiological signals for seizure detection during periods of both wakefulness and sleep.

It is crucial to acknowledge the challenges facing mass adoption of such a technology, which necessitate further exploration. The quality of signals when the ear-EEG device is worn outside clinical settings remains to be assessed. Real-world environments introduce a multitude of variables, from physical activity to environmental noise, that could impact signal quality through the introduction of artifacts. Moreover, the long-term comfort of wearing the earbuds for extended periods during daily activities requires additional research. Understanding user preferences, such as the desire for real-time seizure alerts or hindcasting capabilities, i.e., indicating the probability that a seizure has already occurred, will be pivotal in refining the device’s features and enhancing its user-centric design.

## Conclusions

Our study introduced and validated the efficacy of an innovative ear-EEG system for the long-term monitoring of focal-onset seizures. The system’s discreet and unobtrusive design, complemented by its reliable electrographic signal quality, offers a potentially transformative approach to continuous monitoring of neurological activity outside traditional clinical settings. Notably, this technology proved highly accurate in the capture of temporal lobe seizures, which are prevalent in a significant proportion of epilepsy cases. Furthermore, the device affords a signal that has strong potential for integration with existing algorithms used in the automated analysis of electrographic data, such as real-time seizure detection and forecasting in epilepsy as well as a variety of other neurological applications. An unobtrusive yet reliable wearable device opens the door to routine collection of complementary, longitudinal, remote electrographic evidence that may assist clinicians in making an epilepsy diagnosis, assessing treatment efficacy, and optimizing medication titration.

### Supplementary Information

**Additional file 1.** Algorithm for time alignment of ear-EEG and gold standard EEG. Supplementary Table S1. Patient-wise data acquisition and seizure statistics.

## Data Availability

The datasets analyzed during the current study are available from the corresponding author on reasonable request.

## Abbreviations

*AE*: Adverse Event
*CAPA*: Corrective and Preventive Action
*DC*: Direct Current
*ECG*: Electrocardiogram
*EEG*: Electroencephalography
*EMU*: Epilepsy Monitoring Unit
*FEA*: Fraction of Event Agreement
*FEDA*: Fraction of Event Duration Agreement
*FN*: False Negative
*FP*: False Positive
*IRB*: Institutional Review Board
*PPV*: Positive Predictive Value
*TP*: True Positive
