# Supplementary material for: Using a standalone ear-EEG device for focal-onset seizure detection
Source: Bioelectron Med. 2024 Feb 7;10:4. doi: 10.1186/s42234-023-00135-0 (PMC10848360; doi:10.1186/s42234-023-00135-0)
Supplement: Supplementary file 1 — Additional file 1. Algorithm for time alignment of ear-EEG and gold standard EEG. Supplementary Table S1. Patient-wise data acquisition and seizure statistics. [file 42234_2023_135_MOESM1_ESM.docx]

Supplementary Materials

## Algorithm for time alignment of ear-EEG and gold standard EEG

To align each ear-EEG recording with its corresponding gold standard EEG recording, we analyzed the pulse-train “sync” signals from both modalities. We sampled 100 evenly-spaced windows from the duration of each recording in order to search for drift between the paired signals. Given a maximum anticipated drift of 60 s in either direction, we set the length of each search window to 120 s. Within each search window, we identified peaks from both sync signals and converted them to binary code sequences using 100 ms non-overlapping bins. We then computed the cross-correlation of the code sequences using a 30 s moving window. If the maximal cross-correlation value (i.e., percentage of matched code) was higher than 85%, we identified a "match" and computed the corresponding offset between the two code sequences. To further correct for time offsets at a finer resolution than the code sequence, we computed the actual time differences of all the matching peaks and recorded the median value as the time offset between the two sync signals for that search window. We performed this process for all 100 evenly-spaced search windows and observed a linear drift in time offsets across recording time, likely due to the difference in clock time between the two EEG systems. Therefore, we performed a least-squares linear fit to obtain the initial time offset and the clock drift over the entire recording. Finally, we resampled and shifted the ear-EEG signals to account for both the time offset and clock drift, achieving an error of less than 2-3 ms (i.e. 1-2 samples).

####

#### Supplementary Table S1. Patient-wise data acquisition and seizure statistics.

|  |  | **Total # sessions** | **Total recording time (hr)** | **Mean recording time per session (hr)** | **Total # ground-truth seizures** | **Total # ground-truth seizures**  **included** |
| --- | --- | --- | --- | --- | --- | --- |
| **Reference Modality** | **Patient ID** |  |  |  |  |  |
| **Intracranial EEG** | **1** | 6 | 42.3 | 7.1 | 0 | 0 |
|  | **2** | 2 | 8.5 | 4.2 | 0 | 0 |
|  | **6** | 7 | 42.6 | 6.1 | 2 | 1 |
|  | **7** | 22 | 219.7 | 10 | 2 | 2 |
|  | **8** | 5 | 74 | 14.8 | 16 | 12 |
|  | **9** | 6 | 68.2 | 11.4 | 6 | 5 |
|  | **10** | 5 | 31.7 | 6.3 | 1 | 1 |
|  | **12** | 4 | 32.7 | 8.2 | 0 | 0 |
|  | **20** | 5 | 51.8 | 10.4 | 3 | 3 |
| **Scalp EEG** | **16** | 1 | 14.8 | 14.8 | 0 | 0 |
|  | **20** | 4 | 61 | 15.2 | 3 | 3 |
|  | **23** | 6 | 78.3 | 13 | 3 | 3 |
|  | **26** | 3 | 53.6 | 17.9 | 5 | 4 |
|  | **27** | 3 | 51 | 17 | 1 | 0 |
|  | **28** | 3 | 33.8 | 11.3 | 0 | 0 |
|  | **29** | 4 | 54 | 13.5 | 3 | 0 |
|  | **30** | 8 | 139.8 | 17.5 | 3 | 3 |
|  | **31** | 4 | 67.1 | 16.8 | 6 | 5 |
|  | **32** | 3 | 44.1 | 14.7 | 0 | 0 |
|  | **33** | 1 | 14.3 | 14.3 | 1 | 1 |
|  | **35** | 4 | 71.8 | 17.9 | 1 | 1 |
